# Suppression of host gene expression is associated with latent TB infection: a possible diagnostic biomarker

**DOI:** 10.1038/s41598-024-66486-z

**Published:** 2024-07-07

**Authors:** Ritah Nakiboneka, Nicolò Margaritella, Tonney Nyirenda, David Chaima, Natasha Walbaum, Emmanuel Musisi, Sikwese Tionge, Takondwa Msosa, Marriott Nliwasa, Chisomo L. Msefula, Derek Sloan, Wilber Sabiiti

**Affiliations:** 1 Division of Infection and Global Health, School of Medicine, University of St Andrews, St Andrews, UK; 2 School of Mathematics and Statistics, University of St Andrews, St Andrews, UK; 3 Department of Pathology, Kamuzu University of Health Sciences, Blantyre, Malawi; 4 Pathology Department, Helse Nord Tuberculosis Initiative (HNTI), Kamuzu University of Health Sciences, Blantyre, Malawi; 5 Africa Centre for Public Health and Herbal Medicines (ACEPHEM), Kamuzu University of Health Sciences, Blantyre, Malawi; 6 Adroit Biomedical and Bioentrepreneurship Research Service, Kampala, Uganda; 7 Uganda Virus Research Institute, Entebbe, Uganda

**Keywords:** Diagnostic markers, Tuberculosis, Transcriptomics

## Abstract

The World Health Organization End TB strategy aims for a 90% reduction of tuberculosis (TB) incidence by 2035. Systematic testing and treatment of latent TB infection (LTBI) among contacts of active TB patients is recommended as one of the ways to curtail TB incidence. However, there is a shortage of tools to accurately diagnose LTBI. We assessed the appropriateness of whole blood host transcriptomic markers (TM) to diagnose LTBI among household contacts of bacteriologically confirmed index cases compared to HIV negative healthy controls (HC). QuantiFERON-TB Gold Plus Interferon gamma release assay (IGRA) and reverse-transcriptase quantitative PCR were used to determine LTBI and quantify TM expression respectively. Association between TM expression and LTBI was evaluated by logistic regression modelling. A total of 100 participants, 49 TB exposed (TBEx) household contacts and 51 HC, were enrolled. Twenty-five (51%) TBEx individuals tested positive by IGRA, and were denoted as LTBI individuals, and 37 (72.5%) HC were IGRA-negative. Expression of 11 evaluated TM was significantly suppressed among LTBI compared to HC. Out of the 11 TM, *ZNF296* and *KLF2* expression were strongly associated with LTBI and successfully differentiated LTBI from HC. Paradoxically, 21 (49%) TBEx participants who tested IGRA negative exhibited the same pattern of suppressed TM expression as IGRA positive (LTBI-confirmed individuals). Results suggest that suppression of gene expression underlies LTBI and may be a more sensitive diagnostic biomarker than standard-of-care IGRA.

## Introduction

Latent Tuberculosis Infection (LTBI) is a non-transmissible, asymptomatic state of TB characterised by persistent immune response to stimulation by *Mycobacterium tuberculosis* (MTB) antigens without clinical evidence of active TB disease (ATB)^1^. Reactivation of the bacilli and progression to ATB is estimated to occur at some point in the lifetime of 5–10% individuals with LTBI^2^. This risk is enhanced in immune compromised individuals such as people living with HIV and diabetes, malnourished-, silicosis-, organ transplant-, and renal failure- patients, and those receiving immunosuppressive therapy^3–5^. LTBI reactivation is believed to greatly contribute to new incident cases of ATB globally^6^. The World Health Organization (WHO) End-TB strategy recommends systematic testing and treatment of LTBI to prevent progression to active TB disease and halt transmission^7,8^.

For a long time, LTBI diagnosis has been performed using the tuberculin skin test (TST) and more recently by Interferon Gamma Release Assays (IGRAs), including T-SPOT.*TB* and QuantiFERON-TB Gold Plus (QFT-Plus). These tests are designed to identify an adaptive T-cell memory response to MTB antigens which doesn’t capture the complete spectrum of host immunity towards MTB and depends on innate immunity for its activation. There is *in-vitro* evidence demonstrating that MTB suppresses innate immunity by circumventing phagosome maturation, apoptosis, and induction of autophagy thus hindering its presentation on the major histocompatibility complex (MHC) class II molecule^9,10^. This results into an inert adaptive immune response which may be the cause of negative TST and IGRA results in some individuals with previous exposure to TB index cases^10^. However, negative TST/IGRA results do not necessarily indicate the absence of pathogen or risk of progression to disease. A study by Abubakar et al*.* reported both TST and IGRA negative individuals progressing to ATB^11^ and a WHO review on IGRA performance for ATB diagnosis reported one in four patients with confirmed ATB testing negative for IGRA^12^. Furthermore, for those who test positive, time to resolution of this immune response in absence of live MTB bacteria is unknown^1^. TSTs cross-react with Bacillus Calmette-Guérin (BCG) vaccination and prior exposure to nontuberculous mycobacteria owing to the purified protein derivative (PPD) protein content being highly conserved in *Mycobacterium* species^13^.

In contrast, host gene transcriptional markers (TM) in whole blood more completely reflect both innate and adaptive host response to “newly encountered pathogens" which could offer advantages over IGRA as effective diagnostic biomarkers of LTBI^14^. Although differentially expressed genes have been reported between individuals with active TB disease and those with LTBI or no known TB exposure^15–17^, TM that effectively distinguish LTBI from healthy individuals are incompletely described. Expression profiles described to date in LTBI are from peripheral blood mononuclear cells (PBMCs)^18,19^, sometimes stimulated with the nonspecific PPD^19^, and control groups in clinical studies are often a biased cohort of household contacts with IGRA negative status^18–20^. Focused attention on transcriptomics for ATB progression^21,22^ is important for identification of individuals requiring preventive treatment to halt disease progression, but efforts to achieve this would be enhanced by improved understanding of how the host transcriptome changes throughout the entire course of MTB exposure, including discrimination between gene expression for TB-unexposed healthy controls and those who have developed LTBI.

To this end, we assessed expression of a selected panel of 14 host transcriptomic markers in household contacts of bacteriologically confirmed pulmonary TB cases with IGRA-diagnosed LTBI and TB-exposed but IGRA-negative group and compared this with TM expression in IGRA-negative community controls with no known history of TB exposure. Using logistic regression modelling, adjusting for confounding, multicollinearity, and influential observations, we evaluated which transcriptomic markers were best able to distinguish LTBI from non-exposed IGRA negative healthy controls.

## Materials and methods

### Ethics statement

The study was approved by the College of Medicine Research Ethics committee (COMREC) Malawi under Protocol number P.06/21/3342 and by the University of St Andrews Teaching and Research Ethics Committee (UTREC) under approval code MD15741. All study participants agreed and provided written informed consent before study entry and all study procedures and experiments were conducted in accordance with the relevant guidelines and regulations.

### Study site, and participants

Consenting participants aged 18 years and above were enrolled between 4th Jan and 30th September 2022 from Limbe and Ndirande Primary Health Centres, within Blantyre, Malawi. The study was nested within a larger clinical cohort P.06/21/3342 evaluating transcriptional markers for TB diagnosis and treatment response monitoring.

TB Exposure (TBEx) was defined as being a household contact of a recent bacteriologically confirmed pulmonary TB (PTB) index case. Bacteriological confirmation among index cases was performed by smear microscopy, MGIT culture and Xpert RIF/MTB tests.

PTB participants in the larger clinical cohort were asked to invite their close household contacts to join the study. TBEx individuals who tested positive for QFT-Plus were classified as LTBI while those who were QFT-plus negative were called TBExIGRA-. Healthy controls (HC) were recruited cross sectionally from the same Blantyre community as the TBEx individuals based on the following characteristics: HIV negative status, no known TB exposure, and a negative QFT-Plus (IGRA) test. HC with no known TB exposure who tested QFT-Plus positive were classified as HCIGRA+ .

All laboratory tests were performed at the Kamuzu University of Health Sciences (KUHeS) Pathology department laboratories in Blantyre.

### TB infection diagnosis

QFT-Plus IGRA was used to diagnose TB infection. Briefly for each participant, 1 ml of peripheral venous blood was collected into each of the four special (Nil, TB1, TB2 and Mitogen) TB Gold plus QuantiFERON (Qiagen 622,526) tubes. Blood was adequately mixed by inverting the tubes several times and incubated at 37 °C for 20 h before centrifugation at 3000 g for 15 min to harvest plasma. The harvested plasma was stored at − 80 °C until ELISA was performed according to the manufacturer’s instructions (Qiagen 2018 kit insert). Optical Density (OD) was measured using a microplate reader fitted with a 450 nm wavelength filter and a 620 nm to 650 nm reference filter. Results were interpreted using the QuantiFERON TB Gold test software version 2.71.2.6 provided by Qiagen^23^. An IFN-γ level, after background subtraction, of ≥ 0.35 IU/ml in either TB1 or TB2 QFT-Plus tubes was considered positive. IFN-γ accurate values scoring > 10 IU/ml (QFT-Plus maximum cut-off for high values) were treated as 10 IU/ml.

### Host transcriptomic marker selection for study

Gene marker selection was initially based on frequency of citation in 20 selected signatures namely Anderson42, Anderson 51, BATF2, Berry86, Bloom144, Cai3, Dawany251, Duffy10, Gjoen7, Gliddon3, Gliddon4, Kaforou27, Kaforou44, Costa3, Maertzdorf4, RISK4, Roe3, Suliman2, Sweeney3 and Zak16. Genes that appear in 4 or more signatures were selected to include in the panel. Selection between genes that appeared in 3 signatures was based on biological function and co-expression analysis performed on the STRING website. A gene marker was added to the panel if it was not clustering with the already selected host gene markers or if it had unique biological functions. Other quality control checks including the limit of detection and quantification of the assays were also used to finally confirm the selected host gene marker panel”. An detailed description of the selection procedure is available in methods preprint with eBiomedicine^24^’’.

A total of 14 host TM namely *GBP5, C1QB, KLF2, ZNF296, DUSP3, ASUN, NEMF, PTPRC, DHX29, GBP6, ARG1, GAS6, CD64* and *BATF2* were included in the evaluated panel. Supplementary methods Table 1 summarises the selected marker biological functions. Suitable primers and probe sequencies were designed for all the markers except for Sweeney3 genes (*GBP5*, *DUSP3* and *KLF2*) for which we only designed probe sequencies. Primer sequencies for Sweeney3 genes were those published by Francisco et al*.*^25^.

### Host-gene RT-qPCR assay

From each participant, 2.5 mL of peripheral whole blood was collected into Paxgene tubes and stored at − 80 °C until RNA extraction. Total RNA was isolated using PAXgene blood RNA kit (PreAnalytiX cat.no 762174) as per manufacturer’s recommendation. RNA yield (yield) was measured using Qubit RNA High Sensitivity reagent (Invitrogen Cat. No Q32855) on the Qubit machine version 3.0 (Life technologies)^26^. The host gene reverse-transcriptase quantitative PCR (RT-qPCR) assay was a multiplex platform consisting of target specific primers and probes procured from Eurofins (Germany) and QuantiTect Multiplex RT-PCR kit (Qiagen). RT-qPCR was performed using Rotor-Gene 5plex platform (Qiagen, UK). The final RNA samples used in the RT-qPCR assay were diluted 1:10 in RNase-Free water. Each run consisted of 2 µl of RNA extract and 8 µl of the master mix reagent assayed in duplicates. RT-qPCR cycling conditions described by Honeyborne et al*.*^27^ were adopted. Absolute quantification of the amplified product was performed using standard curves constructed with target oligonucleotide standards of known concentration (copies/μL) procured from Eurofins Genomics, Germany.

### Absolute CD4 quantification

Absolute CD4 cell concentrations in peripheral whole blood collected in EDTA tubes were quantified for participants who were living with HIV using the Alere Pima™ CD4 Machine.

### Statistical analysis

Cycle quantification (Cq) results were converted to concentration using standard curves and recorded as copies/µl. This data was further transformed by conversion of concentration copies/µl to log_10_ copies/µl for skewness reduction. Pearson correlation was performed to describe and measure the relationship between the covariates. Differences between groups were compared using Chi-squared- , Mann–Whitney U- and Kruskal–Wallis- and Dunn's- tests with Bonferroni corrected *p*-values for multiple comparisons. Logistic regression modelling was performed to assess association of transcriptomic marker expression with LTBI vis-a-vis HC individuals. Model selection was carried out among all possible subsets of covariates using the Akaike Information Criterion (AIC) and the likelihood ratio test. Robust models were obtained after considering multicollinearity, linearity of the covariates and influential points, and by subsequently testing the strength of our inferential conclusions using nonparametric alternatives (bootstrap regression). Model goodness of fit was assessed using Hosmer–Lemeshow goodness of fit test. All statistical analyses were conducted in R statistical programming version 4.2.1^28^. Data visualisation was performed using GraphPad Prism (version 10.0.0 Boston, Massachusetts USA) and RStudio 2023.06.1 Build 524 (Posit Software, PBC)^29^.

## Results

### Participant description and baseline characteristics

**Figure 1:**
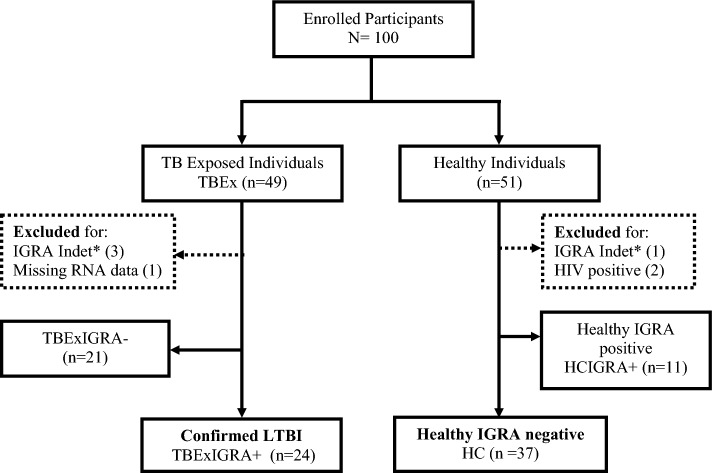
Flow chart of asymptomatic participant categorised based on IGRA results. *Indet = Indeterminate result. Participants in dotted boxes were excluded from further analysis. Participant with missing RNA data was IGRA positive.

A total of 100 asymptomatic individuals (49 TBEx and 51 healthy individuals) were enrolled. Figure 1 illustrates group categorisation based on IGRA results. Results from 4/100 (4%) participants were IGRA test indeterminate thus excluded from further analysis. Compared to healthy participants (n = 48), TBEx participants (n = 45) were older (Median[IQR] age = 36 years [28–42 years] versus 27 years [25–32 years]; *p* = 0.0008, Mann–Whitney U test), yielded less total RNA (Median[IQR] 66.4 ng/µl [44.2–89.4 ng/µl] versus 83 ng/µl [64.8–107.5 ng/µl]; *p* = 0.001, Mann–Whitney U test), and had lower levels of formal education (*p* = 0.006, Chi-squared test).

Twenty-five (51%) TBEx individuals were QFT-Plus positive and classified as LTBI. Eleven (22%) of the healthy participants were IGRA positive and denoted HCIGRA+ . Baseline characteristics of participants in each category are shown in Table 1. Fourteen of the enrolled TBEx individuals were HIV positive (5 LTBI and 8 TBExIGRA-) and their median CD4 count was 501cells/mm^3^ and 509cells/mm^3^ respectively (Table 1). Subsequently, after exclusion of HCIGRA+ and TBExIGRA-, compared to HC (n = 37), LTBI individuals (n = 24) were older (*p* = 0.0002; Mann–Whitney U test), yielded less RNA (*p* = 0.002; Mann–Whitney U test) and were less educated (*p* = 0.006; Chi-squared test). All other variables didn’t differ between groups (Table 1).

**Table 1:** Patient baseline characteristics.

Participant Demographics	Level	LTBI [n = 24]	TBExIGRA- [n = 21]	HC [n = 37]	HCIGRA + [n = 11]
Sex; n [%]	Male	7 [34.7]	8 [38.1]	19 [52.9]	7 [63.6]
Age; years	Median [IQR]	36 [30–42]	36 [28–42]	26 [25–31]	32 [24–39]
Weight; kgs	Median [IQR]	62.5 [– 53–71]	57.3 [53–64]	62 [57.2–68.8]	59.0 [55.6–69]
HIV status; n [%]	Negative	19 [79.2]	13 [61.9]	37 [100]	11 [100]
	Positive	5 [20.8]	8 [38.1]	0	0
CD4 count; cells/mm3	Median [IQR]	501 [459–563]	509 [427–588]	NA	NA
Smoking; n [%]	No	23 [95.8]	21 [100]	32 [86.5]	11 [100]
	Yes	1 [4.2]	0	5 [13.5]	0
Firewood; n[%]	No	22 [91.7]	20 [95.2]	35 [94.6]	11 [100]
	Yes	2 [8.3]	1 [4.8]	2 [5.4]	0
Alcohol; n [%]	No	20 [83.3]	13 [61.9]	27 [73.0]	9 [81.8]
	Yes	4 [16.7]	8 [38.1]	10 [27.0]	2 [18.2]
BCG vaccine; n [%]	Yes	24 [100]	21 [100]	35[94.6]	11 [100]
Can read; n [%]	Yes	23 [95.8]	21 [100]	37 [100]	11 [100]
Work status; n [%]	Employed	13 [54.2]	12 [57.1]	19 [51.4]	7 [63.6]
Marital status; n [%]	Married	18 [75]	18 [85.7]	25 [67.6]	7 [63.6]
Education; n [%]	Secondary	11 [45.8]	13 [61.9]	23 [62.2]	8 [72.7]
	Tertiary	4 [16.7]	2 [9.5]	12 [32.4]	2 [18.2]
RNA yield; ng/µl	Median [IQR]	59 [44–84.6]	72 [52.4–112]	91.2 [72.4–117]	71 [59–83]
IGRA; n [%]	Positive	24 [100]	0	0	11 [100]
	Negative	0	21 [100]	37 [100]	0

### Comparative analysis of interferon gamma release level and TM expression in LTBI compared to HC participants

**Figure 2:**
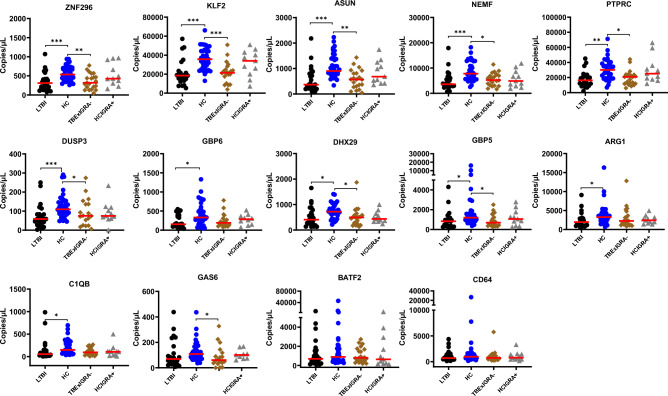
Whole blood expression profiles among the enrolled participants. Host gene markers expression profiles among LTBI (black), HC (blue), TBExIGRA- (brown) and HCIGRA + (grey) individuals is shown. Red lines indicate the median. Statistical comparison was performed using the Kruskal–Wallis test and Dunn's Test with Bonferroni corrected *p*-values for multiple comparisons. * =  < 0.05, ** =  < 0.01, and *** =  < 0.001.

Using Mann Whitney U test, we assessed whether TM expression was different in LTBI participants compared to HC. Sixty-one participants (24 LTBI and 37 HC) were analysed. Mean [SD] interferon gamma (IFN-γ) level of the LTBI individuals was 4.2 ± 3.6 IU/ml and 4.03 ± 3.5 IU/ml for QFT-Plus tubes TB1 and TB2 respectively. Correspondingly, low levels of TM *ZNF296* (310.5 copies/µl vs. 535 copies/µl)*, KLF2* (18625 copies/µl vs. 35650 copies/µl), *ASUN* (364.7 copies/µl vs. 902.5 copies/µl), *NEMF* (3577.5 copies/µl vs. 7780 copies/µl), *PTPRC* (16350 copies/µl vs. 30100 copies/µl), *DUSP3* (58.7 copies/µl vs. 110.5 copies/µl), *GBP6* (155.5 copies/µl vs. 332 copies/µl), *DHX29* (407.8 copies/µl vs. 719 copies/µl), *GBP5* (878 copies/µl vs 1195.5 copies/µl), *ARG1* (1952.5 copies/µl vs. 3265 copies/µl), and *C1QB* (56 copies/µl vs 149.5 copies/µl) were observed in LTBI compared to HC respectively using Kruskal–Wallis test and Dunn's Test for multiple comparisons. Figure 2 illustrates significant group comparisons.

### Robust modelling confirmed suppressed expression marker levels were significantly associated with LTBI

Robust logistic models were obtained by first evaluating collinearity, possible transformations of the covariates and the presence of influential observations on a first set of logistic regression models where the effect of the host gene markers on the probability of LTBI was corrected by age and yield. Influential point analysis detected two observations with strong influence (statistic > 10) on the regression parameters limiting the performance of the fitted models (supplementary analysis 1). These 2 observations were assessed (see discussion) and removed. Model selection among multiple subsets of the covariates was carried out using the AIC and likelihood ratio tests. Our results showed that models including the suppressed expression of one of the host gene markers *ZNF296* (*p* = 0.008), *KLF2* (*p* = 0.012), or *DUSP3* (*p* = 0.037), significantly discerned LTBI from HC individuals, after controlling for age and yield (Table 2 and supplementary analysis 2). The regression equations for host gene marker *ZNF296* showed that, holding constant age and RNA yield, a decrease of 91 copies/µl (0.1log_10_ decrease) in expression level of an individual from the median of 444 copies/µl was associated with 48% increase in the odds of having LTBI. The same decrease in the log expression marker *KLF2* (6458 copies/µl) and *DUSP3* (19 copies/µl) from the median log expression level of 31,400 copies/µl and 92 copies/µl respectively, was associated with a 46% and 35% increase in the odds of LTBI. Reduced expression levels of host gene markers *ASUN, PTPRC*, and *GBP5* also showed a trend in association with LTBI although this was not statistically significant possibly due to the small sample size (supplementary analysis 2).

**Table 2:** Showing regression estimates for the significant expression markers.

Variable (log10)	Estimate	Std. Error	z value	Pr( >|z|)
*ZNF296*	− 6.558	2.502	− 2.621	0.009**
*KLF2*	− 6.244	2.477	− 2.521	0.012*
*DUSP3*	− 4.308	2.075	− 2.076	0.038*

### Models with ZNF296 and KLF2 best fit data and are reproducible for LTBI classification

The Likelihood ratio test and delta AIC showed that inclusion of the expression marker of either *ZNF296* or *KLF2* to the model with age and yield alone significantly improved the model fit with a *p*-value of 0.0009 and 0.0036 respectively and a noticeable reduction in the AIC (Table 3) thus TM preceded all the rest and were further evaluated for robustness. Model check analysis confirmed that these TM met the linearity assumptions (Supplementary Analysis 3). Hosmer–Lemeshow (HL) goodness of fit test for the best models including ZNF296 and KLF2 showed no evidence of poor fit for all risk categories (supplementary Analysis 4). Lastly, bootstrapping analysis revealed that the bootstrap standard errors of the estimates of *ZNF296* and *KLF2* were similar to those obtained in the standard logistic regression model and the coefficient estimates were within the 95% CI of the bootstrap estimates (Supplementary Analysis 5). These results are highly suggestive that the role of suppressed expression levels of the host gene markers ZNF*296* and *KLF2* in LTBI detection could be confirmed in a larger population.

**Table 3:** Likelihood Ratio test and AIC (Akaike Information Criterion) results.

Models	Likelihood ratio test				AIC	Delta AIC^#^	
	Deviance Resid	Df Resid	Dev	Pr(> Chi)		Model age—model X	Model age + yield—model X
	NULL	58	77.94				
Age	21.72	57	56.22	3.162e-06***	60.22		
Age + yield	8.92	56	47.3	0.002825**	53.30	6.92	
Age + yield + *ZNF296*	10.90	55	36.4	0.0009606***	44.40	15.82	**8.90**
Age + yield + *KLF2*	8.44	55	38.86	0.003670**	46.86	13.36	**6.44**
Age + yield + *DUSP3*	6.11	55	41.19	0.013421*	49.19	11.03	4.11
Age + yield + *ASUN*	4.78	55	42.53	0.028842*	50.53	9.69	2.78
Age + yield + *GBP5*	3.65	55	43.65	0.056105	51.65	8.57	1.65
Age + yield + *PTPRC*	3.11	55	44.19	0.077857	52.19	8.03	1.11
Age + yield + *NEMF*	2.98	55	44.32	0.084195	52.32	7.90	0.98
Age + yield + *GBP6*	1.71	55	45.60	0.191	53.60	6.63	− 0.29
Age + yield + *C1QB*	1.28	55	46.02	0.258	54.02	6.20	− 0.72
Age + yield + *ARG1*	0.60	55	46.70	0.438	54.70	5.52	− 1.40
Age + yield + *GAS6*	0.52	55	46.78	0.469	54.78	5.44	− 1.48
Age + yield + *DHX29*	0.08	55	47.22	0.778	55.22	5.00	− 1.92
Age + yield + *CD64*	0.01	55	47.30	0.931	55.30	4.93	− 1.99
Age + yield + *BATF2*	0.31	55	46.99	0.578	54.99	5.23	− 1.69

*Significant *p*-value for likelihood ratio test.

^#^A change in AIC of ≥ 5 is considered noticeable.

Values in bold refer to significant predictive models.

### Similar gene expression profiles were seen in TBExIGRA- individuals as in LTBI

The TM expression levels among TBExIGRA- (n = 21) and HCIGRA+ (n = 11) participants were profiled. Mean [SD] IFN-γ levels were 0.03 ± 0.08 IU/ml and 0.04 ± 0.08 IU/ml for TBExIGRA- and 3.2 ± 3.7 IU/ml and 2.6 ± 2.8 IU/ml for HCIGRA+ individuals for QFT-Plus tubes TB1 and TB2 respectively.

TBExIGRA- expression profiles were significantly suppressed for host gene markers *ZNF296, KLF2, ASUN, NEMF, PTPRC, DUSP3, DHX29, GBP5* and *GAS6* compared to expression among HC (Refer to Fig. 2). No difference in expression profiles among TBExIGRA- and LTBI participants was observed. Similarly, expression levels in HCIGRA + didn’t differ from expression profiles among either HC or expression among LTBI individuals although this expression profile was slightly suppressed as in LTBI (Fig. 2).

### Suppressed expression profiles among both HIV- Negative and Positive TB Exposed contacts

**Figure 3:**
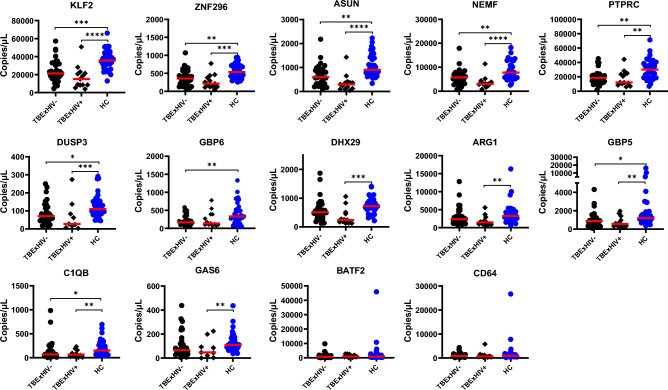
TB exposure is associated with a downregulated pattern of expression profiles. Scatter plots of median show that irrespective of IGRA status and HIV status, TB exposure (black dots) affects gene expression compared to no known history of TB exposure–HC individuals (blue dots). TBExHIV- included 19 LTBI and 13 TBExIGRA- participants while TBExHIV + included 5 LTBI and 8 TBExIGRA- participants. Statistical comparison was performed using Kruskal–Wallis test and Dunn's Test with Bonferroni corrected *p*-values for multiple comparisons. *Denotes *p* < 0.05, **denotes *p* < 0.01, ***denotes *p* < 0.001 and ****denotes *p* < 0.0001.

To verify whether the TM expression pattern among TBEx household contacts was not a result of HIV co-infection, we compared expression profiles of TBEx HIV negative (32/45) and TBEx HIV positive (13/45) participants to HC (n = 37), irrespective of IGRA status. No significant difference in gene expression profiles was observed between TBEx with either HIV negative (TBExHIV-) or HIV positive (TBExHIV+) status for all gene markers (Kruskal–Wallis test and Dunn's Test with Bonferroni corrected *p*-values for multiple comparisons). Compared to HC, gene expression in TBExHIV- participants was significantly suppressed for host TM *KLF2, ZNF296, ASUN, NEMF, PTPRC, DUSP3, GBP6, GBP5* and *C1QB*. Similarly, gene expression among TBExHIV+ participants was suppressed for host gene TM *KLF2, ZNF296, ASUN, NEMF, PTPRC, DUSP3, GBP5*, *C1QB, DHX29, ARG1*, and *GAS6* (Fig. 3).

### Gene expression strongly positively correlated

**Figure 4:**
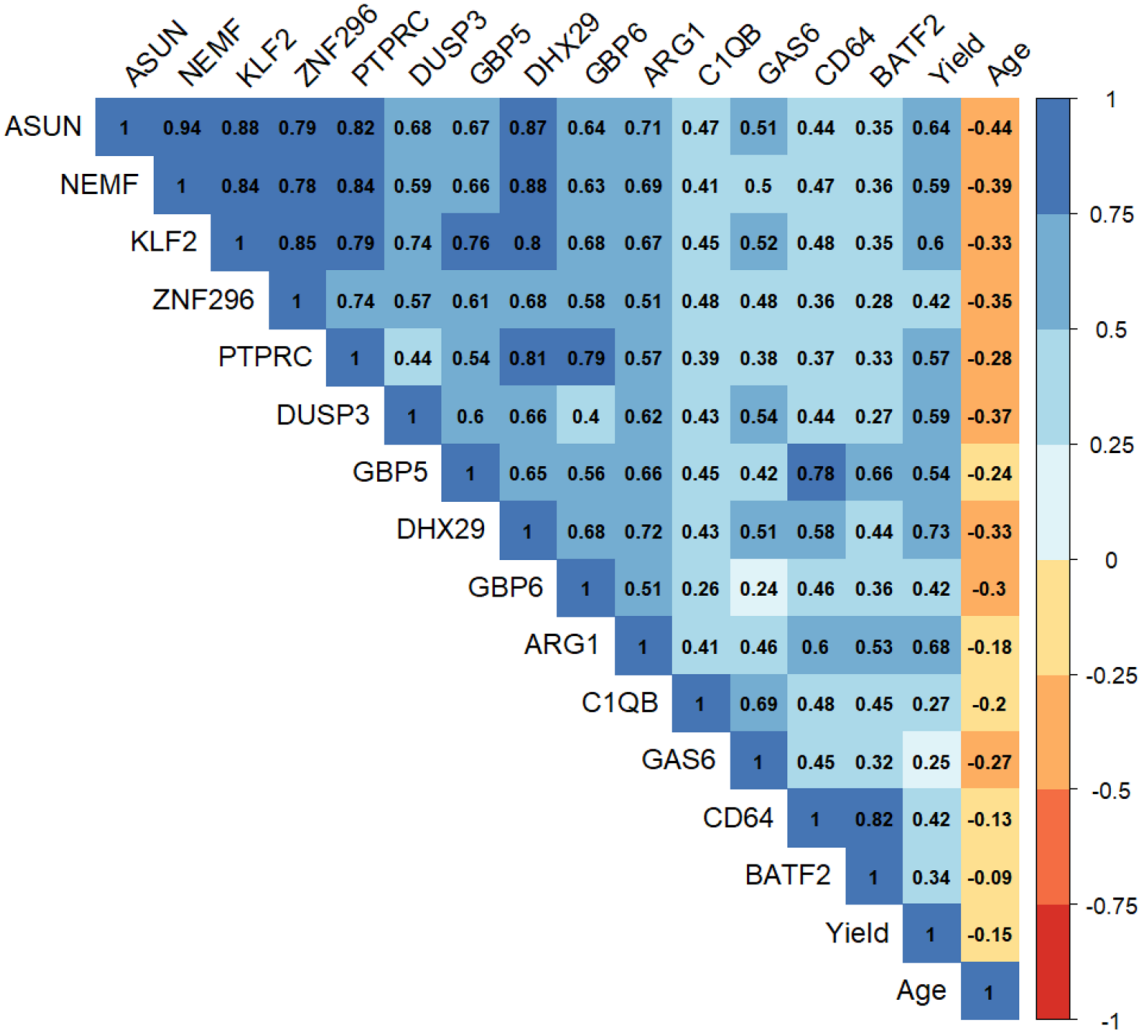
Correlation matrix showing evaluated variable association patterns. The colour scale indicates the degree of correlation.

Finally, correlation between the evaluated Transcriptomic markers and the covariates age, and yield was assessed. Strong positive linear correlation was observed between several variables. The best performing host transcriptional genes *ZNF296* and *KLF2* were positively correlated with each other and with *ASUN*, *NEMF*, *PTPRC*, and *DHX29* (Fig. 4). Moderate positive association was observed between Transcrptomic markers *DUSP3*, *GBP5*, *GBP6* and *ARG1* with each other and with the previously mentioned strongly associated markers. *CD64* was positively correlated with *BATF2* and *GBP5*. A moderate relationship was seen between yield and some trancriptomic markers namely *DHX29*, *ARG1*, *ASUN*, *KLF2*, *DUSP3*, *NEMF* and *GBP5*. Age was weakly negatively correlated with most of the evaluated host gene markers. No association between age and yield was observed.

## Discussion

The rationale for this study was to evaluate a plausible panel of host transcriptional markers for LTBI diagnosis. Using a cohort of 61 individuals and modelling approach that accounted for confounding, multicollinearity, influential observations and violations of the normality and linearity assumptions, two transcriptomic markers; *ZNF296* and *KLF2,* adjusted for the effect of age and RNA yield, exhibited the highest ability in differentiating LTBI from HC individuals. Implying that the two host gene markers were valuable in LTBI classification hence plausible for utility in LTBI diagnosis. The analyses increased the likelihood that our results will be reproducible and confirmed in future larger studies.

Both ZNF296 and KLF2 belong to the same family of Zinc finger DNA binding proteins and function as regulators of gene expression^30–33^. This could explain the strong significant positive association that these two gene markers had with each other. Limited literature is available for ZNF296 although it is predicted to be involved in positive regulation of transcription by RNA polymerase II^32^. On the other hand, KLF2 has been extensively studied^30,31^ and reported as a regulator of several inflammmatory genes and cytokines^31^. This role is accomplished through regulating the transcriptional activity of Nuclear factor kappa-light-chain-enhancer of activated B cells (NF-κB) through competitive interaction with PCAF (chromatin modulators p300/cyclic adenosine monophosphate response element binding protein (CBP)-associated factor)^31^. MTB secreted tyrosine phosphatase (PtpA) prevents activation of the NF-κB, kinases Jnk and p38 signalling pathways that are crucial in innate immunity activation^9^, so suppressed levels of the immune regulator genes could reflect MTB’s interference with the host immune regulatory system.

Two LTBI individuals with relatively high expression markers were excluded from further modelling analysis for LTBI diagnosis. Proximity with a bacteriologically confirmed TB case is a critical risk factor for TB infection and progression to ATB and 5–10% of those who get infected are reported to progress to active TB disease^2^. It is possible that the two individuals, (8.3% (2/24)), were possible progressors and would have benefited from further evaluation. Zak et al. reported a 16 transcriptional signature with 71.2% sensitivity for discriminating progressors from non-progressors, 6 months before diagnosis of these individuals with active TB and these progressors had upregulated levels of host gene markers^21^. Another similar study by Burel et al*.* showed that LTBI individuals at risk of ATB progression shared a similar upregulated transcriptome (21 genes) like ATB compared to non-progressing LTBI^34^; further suggestive of progression likelihood of the 2 individuals and demonstrating TM’s ability to possibly predict TB disease progression. However, the study captures a snapshot in time and was not designed to provide insights into TB progression.

Differentially expressed markers in LTBI compared to HC participants also exhibited a downregulated pattern for other host gene markers namely *ASUN, NEMF, PTPRC, DUSP3, GBP6, DHX29, GBP5, ARGI* and *C1QB*. These gene markers are also involved in immune response regulation^35,36^, transcription regulation^37,38^ and cell cycle control^39^. MTB has been shown to alter host cell physiology through release of effector molecules called nucleomodulins that target the nucleus and hijack nuclear processes including transcription, chromatin reorganisation, and posttranslational modification^10^. As a result, MTB is reported to suppress expression of a range of host genes^9^, which could explain these similar findings. Similar pattern of suppression was reported by Lee et al*.* in Taiwan. They reported 111 host gene markers suppressed in LTBI compared to HC^18^. Bade et al*.* also reported significant deregulation of macrophage host response genes in-vitro when they infected macrophages with the H37Rv strain of MTB for 1 h and 3 hours^40^ possibly indicative of early/ latent stage of infection.

Of note, we have shown that individuals with LTBI had low levels of RNA yields after extraction compared to HC participants. RNA yield was measured with the Qubit RNA High Sensitivity reagent^26^. This assay measures total RNA, ribosomal (rRNA) and large messenger (mRNA). The RT-qPCR assay reverse transcribes mRNA to complementary DNA (cDNA), amplifies and quantifies cDNA in the sample. Absolute quantification methods will quantify the amount of amplified product which is directly proportional to the amount of starting mRNA target in the sample. Low levels of expressed gene markers mirror the low amount of target (mRNA) present in the initial sample. All this may correspond to MTB hijack of the cell cycle, transcriptional and translational machineries of the cells.

Furthermore, similar low expression levels in TBEx IGRA negative individuals as in LTBI individuals suggests that IGRA test is underdiagnosing LTBI among TB exposed participants. No prior study has specifically evaluated this phenomenon, although Kaul et al*.*^20^ showed no differences in expression for *ASUN, NEMF* and *GBP5,* genes that we also evaluated, among IGRA positive vs IGRA negative household contacts in India. The control group used in the Indian cohort were exposed household IGRA negative individuals and their assay diagnostic performance was very low for a combination of markers^20^. As stated above, MTB secreted protein tyrosine phosphatase (PtpA) targets the vacuolar-H^+^-ATPase machinery in the host cytosol and inhibits phagosome acidification and the NF-κB pathway^9,10,41,42^. The NF-κB is responsible for activation of the innate immune system through controlling DNA transcription. NF-κB inhibition causes suppression of host inflammatory immune response^10^ and delays activation of the adaptive immune response. This could explain the negative IFN-γ release assay results in TBExIGRA-, that possibly T-cells had not been primed by the infection but TBExIGRA- individuals may be LTBI and that IGRA result is a false negative.

Previous literature has called household contacts who remain TST/IGRA- negative after repeated exposure to ATB as “resisters” or “infection resisters” implying that these individuals remain uninfected or have enhanced host immunity to rapidly clear their infection^43^. Studies in this cohort of individuals have been difficult with others arguing that resisters might simply not have had sufficient exposure to an infectious dose^43^. We have shown that TBExIGRA- individuals possibly “resisters” are indeed having suppressed transcriptional profile as LTBI an indication of possibly sufficient exposure. Hence, transcriptional studies are of much more biological significance if correlates of protection among resisters are to be further explored. Moreover, suppressed levels of expression profiles seen in both HIV negative and HIV positive TBEx individuals compared to HC further support the specificity of the downregulated pattern of host gene marker expression in TBEx as being a result of TB exposure rather than HIV co-infection.

Finally, we acknowledge some limitations of our work. TB exposure among household contacts was self-reported, timing and duration of exposure was unknown, overall group category sample sizes were small and since only a small proportion (fourteen) of profiled expression markers were selected from previous literature, there is a possibility of having missed out important markers. In addition, the study was undertaken in a specific population and generalizability in different ethnic groups was not determined. However, the evaluated gene markers were initially discovered in different genetic backgrounds, and those frequently reported as highly promising for TB infection diagnosis were chosen. Therefore, TM generalization to different population groups might not be a problem. An evaluation of how this signal in LTBI compares with expression in ATB is also not reported here. This is being undertaken in parallel and will be reported separately. Nevertheless, the study had several methodological strengths which include the construction of models that account for a possible confounding effect of age and RNA yield, the detection of influential observations with a negative impact on the stability of the model parameters, and a careful assessment of the model assumptions and their possible violations. All these analyses helped ensure that our results are more likely to be confirmed in larger samples and hence that the gene expression TM identified are indeed promising candidates for the development of diagnostic biomarkers for detection of LTBI in the general population. Result validation in a larger clinical cohort with well characterised participant groups, well mapped out exposure duration, and comprehensive profiling of the transcriptional markers will be valuable.

In conclusion, our results support findings of MTB infection interfering with host innate immune response activation seen by the suppressed expression profiles of TM. The suppressed expression pattern was observed in both IGRA positive and a proportion of IGRA negative TB exposed individuals, suggesting that host gene expression markers are more sensitive in detecting LTBI than IGRA. Future larger studies will enable designing of suitable predictive models, including proper description of the clinical significance of these host gene expression markers and gene expression thresholds for LTBI diagnosis.

### Supplementary Information

Supplementary Information.

## Data Availability

All data generated or analysed during this study are available on the University of St Andrews OneDrive and is accessible upon request from the corresponding author and meeting the ethical requirements as per consent received from study participants. Please contact ws31@st-andrews.ac.uk for data access.
