# Supplementary material for: Suppression of host gene expression is associated with latent TB infection: a possible diagnostic biomarker
Source: Sci Rep. 2024 Jul 7;14:15621. doi: 10.1038/s41598-024-66486-z (PMC11228037; doi:10.1038/s41598-024-66486-z)
Supplement: Supplementary file 1 — Supplementary Information. [file 41598_2024_66486_MOESM1_ESM.pdf]

Title: Suppression of host gene expression is associated with latent TB infection: a possible diagnostic biomarker.

Ritah Nakiboneka[1,3,4,5,7]; Nicolò Margaritella[2]; Tonney Nyirenda[3]; David Chaima[3]; Natasha Walbaum[1]; Emmanuel Musisi[1,6]; Sikwese Tionge[4]; Takondwa Msosa[4]; Marriott Nliwasa[3,4]; Chisomo L. Msefula[3,4,5]; Derek Sloan[1]; Wilber Sabiiti[1*].

Supplementary methods Table 1: Summary of the evaluated host gene markers and their biological functions

Gene symbol and Publications	Gene Full Name	Biological function
GBP5 [15,17,21,41–45]	Guanylate-binding protein family member 5	*GBP5* is an interferon gamma (IFN-γ) induced gene that plays a role in innate immunity and inflammation [46]. Its encoded protein activates the NLRP3 inflammasome assembly.
GBP6 [15,17,41,47–49]	Guanylate-binding protein family member 6	*GBP6* is an IFN-γ induced gene that is reported to offer protection from several pathogens [50]. Suppressed levels of this gene have been linked to poor cell differentiation [51].
C1QB [15,17,41,48,49,52]	Complement C1q B Chain	Is involved in innate and adaptive immunity where it activates the classical complement system. The complement cascade part of the immune system enhances the ability of phagocytic cells and antibiotics to kill microbes, clear damaged cells, and induce inflammation [53]. *C1QB* proteins are involved in T cell activation following antigen presentation.
CD64 [15,17,21,42,47,48]	Cluster of Differentiation 64	Also known as *CD64A* - Fc fragment of IgG receptor Ia (*FCGR1A*) and *CD64b*- Fc fragment of IgG receptor Ib (*FCGR1B*) *CD64* is involved in IFN-γ signalling pathway and is induced by IFN-γ [54]. It encodes a high-affinity Fc-gamma receptor protein that binds IgG with high affinity [55]. *CD64* is highly expressed on resting monocytes, macrophages [56], and neutrophils [57].
GAS6 [15,17,47,58]	Growth arrest specific 6	It was originally found as a gene upregulated by growth arrested fibroblasts. It's Involved in stimulation of cell proliferation. This gene is frequently overexpressed in many cancers and has been implicated as an adverse prognostic marker [59]
BATF2 [15,17,21,43,49]	Basic Leucine Zipper Transcription Factor 2	*BATF2* participates in the immune system through controlling differentiation of lineage specific cells [60].
DUSP3 [17,44,47]	Dual specificity phosphatase 3	*DUSP3* encodes a protein that is responsible for inactivating of target kinases and regulating mitogen-activated protein (MAP) kinase members [32]. The MAP kinase members are associated with cellular proliferation and differentiation. Thus, *DUSP3* is involved in cellular proliferation and differentiation regulation [32].
ASUN [18,61]	Asunder spermatogenesis regulator Also known as Integrator complex subunit 13 (INTS13)	Is a cell cycle regulator that acts in the nucleus in connection with other integrator components to mediate recruitment of dynein to the nucleus envelope [36].
DHX29 [18,61]	DExH-box helicase 29	Encoded protein functions in translation initiation [62] and is specifically required for ribosomal scanning across stable mRNA during initiation codon selection [63]
KLF2 [44]	Krüppel-like Factor 2	Regulates several inflammatory genes and cytokines through regulating the transcriptional activity of NF-κB through competitive interaction with P300/CBP-associated factor (PCAF) [28]
ZNF296 [47,49]	Zinc finger protein 296	Is predicted to be involved in positive regulation of transcription by RNA Polymerase II and spermatogenesis [29]
PTPRC [18,61]	Protein tyrosine phosphatase, receptor type, C	Also known as *CD45* is an essential regulator of T- and B-cell antigen receptor signalling [33]. Protein Tyrosine Phosphatases are known to be signalling molecules that regulate a variety of cellular processes including cell growth, differentiation, mitosis, and oncogenic transformation [64].
NEMF [18,61]	Nuclear export mediator factor	Is part of the ribosome quality control complex, a ribosome-associated complex that mediates ubiquitination and extraction of incompletely synthesized nascent chains for proteasomal degradation [34].
ARG1 [47,49]	Arginase	The *ARG1* gene provides instructions for producing the enzyme arginase. Arginase enzyme participates in the urea cycle, a series of reactions that occurs in liver cells. Increased expression of *ARG1* induced by MTB reduces the production of nitric oxide required for MTB killing [65]. Has been reported to be expressed in increased amounts in the granuloma of TB patients [66].

Supplementary analysis 1: Influential point (DifChiSQ) analysis

Summary table of the influential points for all models tested. Observations with a score of >5 and >10 are reported.

Gene marker	DifChiSq statistic > 5	DifChiSq statistic > 10
1. *ZNF296*	**19, 24**	**19, 24**
2. *PTPRC*	9, **19, 24**	**19**
3. *KLF2*	5, **19, 24**	**19, 24**
4. *GBP6*	9, **19, 24**	0
5. *ASUN*	4, 5, 9, **19**	**19**
6. *NEMF*	5, 9, **19**	**19**
7. *DUSP3*	5, 9, **19, 24**	**24**
8. *GAS6*	5, **19**, 20, **24**	0
9. *GBP5*	5, 9, **19, 24**	**24**
10. *C1QB*	5, 20, **24**	0
11. *ARG1*	5, **19**, 20, **24**	0
12. *DHX29*	**19**, 20, **24**	0
13. *CD64*	**19**, 20, **24**	0
14. *BATF2*	**19**, 20	0

Supplementary Analysis 2: Logistic regression for each gene expression marker corrected for Age + Yield.

ZNF296

```
Coefficients:
            Estimate    Std. Error.  z value    Pr(>|z|)
(Intercept) 12.59263    6.76690      1.861      0.06276 .
Age          0.18780    0.06688      2.808      0.00499 **
Yield       -0.02307    0.01416     -1.629      0.10340
ZNF296      -6.55833    2.50249     -2.621      0.00877 **
```

KLF2

```
Coefficients:
              Estimate Std. Error z value Pr(>|z|)
(Intercept) 22.004445  10.663263   2.064  0.03906 *
Age           0.181945   0.062349   2.918  0.00352 **
Yield        -0.009706   0.014790  -0.656  0.51165
KLF2         -6.244004   2.476872  -2.521  0.01170 *
```

DUSP3

```
Coefficients:
            Estimate Std. Error z value Pr(>|z|)
(Intercept)  2.75423    4.05583   0.679  0.49709
Age          0.19326    0.06700   2.884  0.00392 **
Yield       -0.01435    0.01477  -0.972  0.33120
DUSP3       -4.30851    2.07504  -2.076  0.03786 *
```

ASUN

```
Coefficients:
             Estimate Std. Error z value Pr(>|z|)
(Intercept)  6.08850    5.86027   1.039   0.2988
Age          0.16679    0.06785   2.458   0.0140 *
Yield       -0.01183    0.01600  -0.739   0.4597
ASUN        -3.85017    1.97943  -1.945   0.0518 .
```

PTPRC

```
Coefficients:
            Estimate Std. Error z value Pr(>|z|)
(Intercept)  8.29761    7.96764   1.041  0.29768
Age          0.20138    0.06681   3.014  0.00258 **
Yield       -0.02165    0.01504  -1.440  0.14996
PTPRC       -3.10629    1.86738  -1.663  0.09622 .
```

NEMF

```
Coefficients:
             Estimate Std. Error z value Pr(>|z|)
(Intercept)  6.15364    7.11789   0.865  0.38730
Age          0.18838    0.06734   2.797  0.00515 **
Yield       -0.01891    0.01539  -1.228  0.21927
NEMF        -2.95622    1.86340  -1.586  0.11263
```

GBP5

```
Coefficients:
            Estimate Std. Error z value Pr(>|z|)
(Intercept)  1.94224    4.39863   0.442  0.65881
Age          0.21210    0.06513   3.256  0.00113 **
Yield       -0.01989    0.01504  -1.322  0.18602
GBP5        -2.53349    1.50473  -1.684  0.09224 .
```

GBP6

```
Coefficients:
             Estimate Std. Error z value Pr(>|z|)
(Intercept) -1.84614    2.99697  -0.616  0.53789
Age          0.21200    0.06693   3.167  0.00154 **
Yield       -0.02824    0.01445  -1.955  0.05058 .
GBP6        -1.31439    1.08122  -1.216  0.22412
```

DHX29

```
Coefficients:
            Estimate Std. Error z value Pr(>|z|)
(Intercept) -3.36997    5.46804  -0.616  0.53769
Age          0.22127    0.06868   3.222  0.00127 **
Yield       -0.03152    0.01988  -1.586  0.11277
DHX29       -0.58448    2.07421  -0.282  0.77811
```

ARG1

```
Coefficients:
             Estimate Std. Error z value Pr(>|z|)
(Intercept) -0.22776    6.11330  -0.037 0.970280
Age          0.22150    0.06532   3.391 0.000696 ***
Yield       -0.02585    0.01807  -1.430 0.152718
ARG1        -1.53070    1.98337  -0.772 0.440254
```

C1QB

```
Coefficients:
            Estimate Std. Error z value Pr(>|z|)
(Intercept) -3.66667    2.22831  -1.645  0.09987 .
Age          0.21626    0.06576   3.289  0.00101 **
Yield       -0.03176    0.01386  -2.291  0.02198 *
C1QB        -0.59814    0.53644  -1.115  0.26484
```

GAS6

```
Coefficients:
             Estimate Std. Error z value Pr(>|z|)
(Intercept) -3.90153    2.34441  -1.664  0.09608 .
Age          0.21722    0.06692   3.246  0.00117 **
Yield       -0.03265    0.01419  -2.301  0.02141 *
GAS6        -0.47151    0.66248  -0.712  0.47663
```

BATF2

```
Coefficients:
            Estimate Std. Error z value Pr(>|z|)
(Intercept) -5.74307    2.70646  -2.122 0.033839 *
Age          0.23151    0.06721   3.445 0.000572 ***
Yield       -0.03897    0.01538  -2.534 0.011267 *
BATF2        0.36705    0.68572   0.535 0.592457
```

CD64

```
Coefficients:
             Estimate Std. Error z value Pr(>|z|)
(Intercept) -4.52905    3.83578  -1.181 0.237708
Age          0.22635    0.06646   3.406 0.000659 ***
Yield       -0.03502    0.01576  -2.222 0.026282 *
CD64        -0.10766    1.24191  -0.087 0.930919
```

Supplementary analysis 3: Linearity Assumptions evaluations

Residual linearity analysis

Further model check analysis to confirm that linearity assumptions were met was performed. Both quadratic and smoother-based curves were fitted to the residual plots. Both the model with *ZNF296* (Model 1) and *KLF2* (Model 2) met the assumptions of linearity meaning that the covariates response is explained in a linear form since they align on the link scale thus no transformation of the data was required. Also, the test statistics for their coefficients were small and nonsignificant.

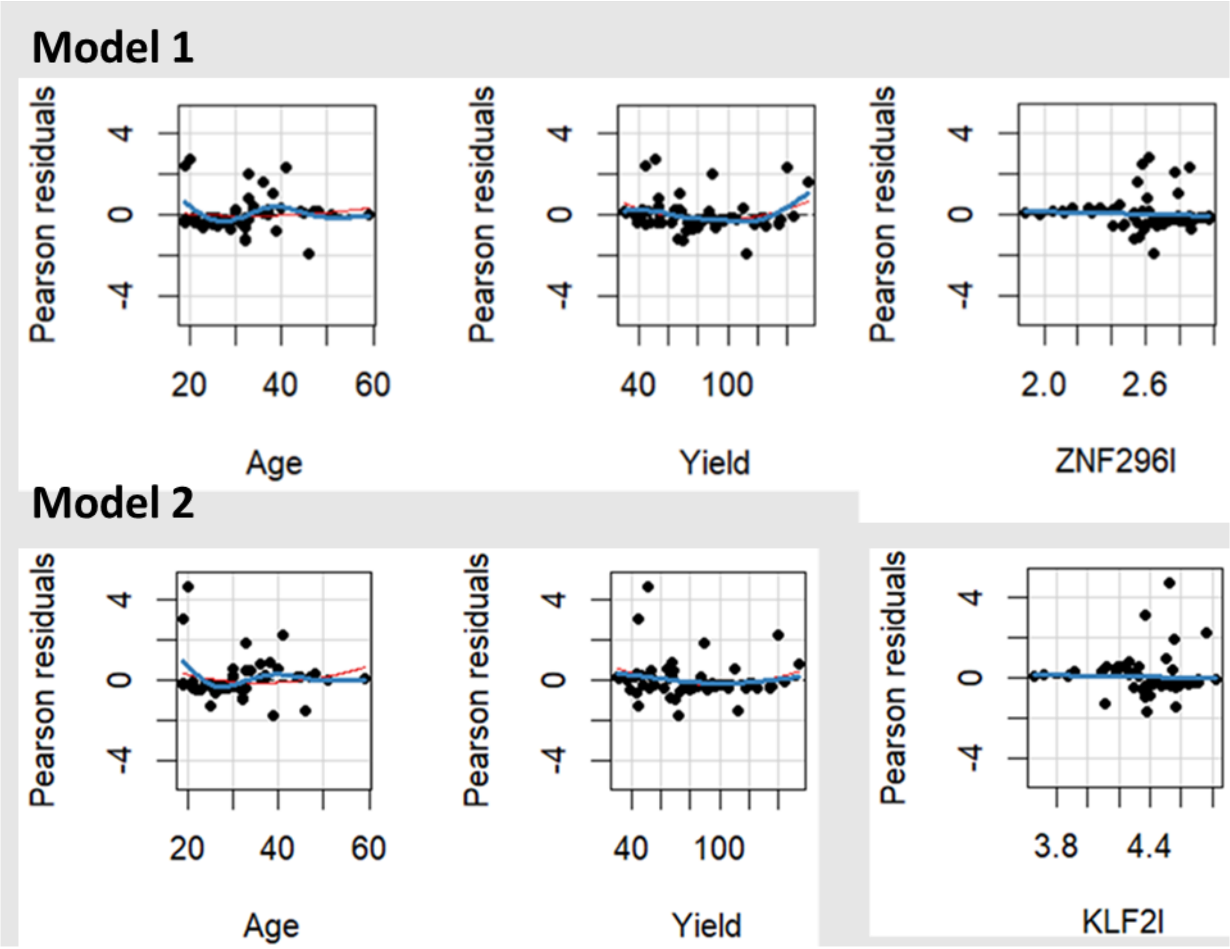

Supplementary figure 3A: Residual plots for the fitted models showing residue linearity assumption for the best-fit models. The red curve shows the fitted quadratic term and blue curve shows the fitted smoother-based term.

Pearson Residual versus Fitted Analysis

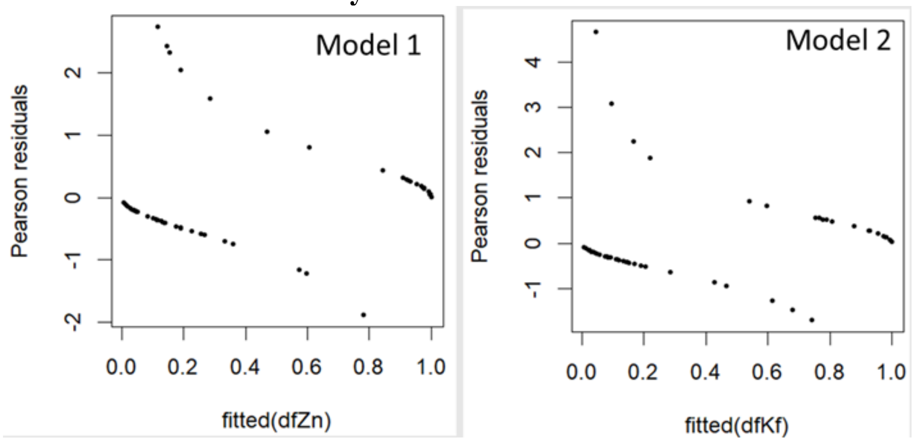

Supplementary Figure 3B: Residual vs fitted analysis. Residuals for model 1 are all between -2 and 2.5 while a couple of outliers can be seen in model 2.

Mean- Variance Relationship Analysis

A Logistic regression model should show more uncertainty around fitted probabilities of 0.5 and lower uncertainty at the two extremes (0 and 1). To check this assumption, we plotted the mean fitted values vs residual variance, after the data were grouped in 5 bins (according to the fitted values) and both the fitted values mean and the residuals variance within each bin were measured.

A correct mean-variance relationship is shown for model 1. The higher variation in the first bin of model 2 is driven by the outlier still present in this analysis (subjects 5). This subject had very high expression levels of the host gene marker and was also possibly transitioning to subclinical/ ATB disease. Nonetheless, they were not considered highly influential on the model parameters (**see supplementary analysis 1**) and therefore were kept in the model.

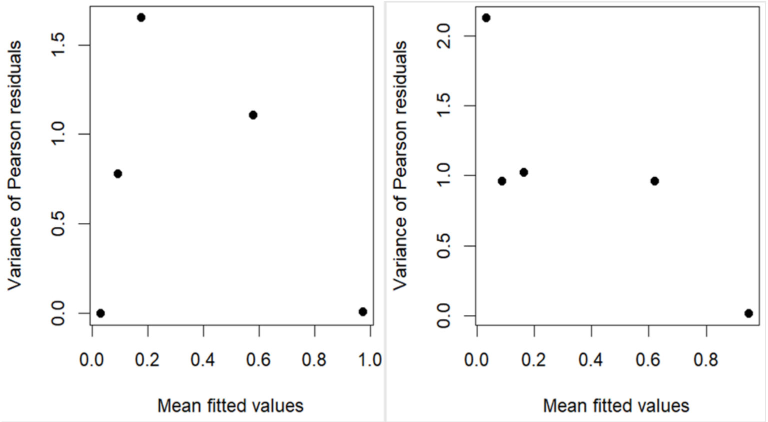

Supplementary Figure 3C*: Mean-variance relationship for the fitted models.*

Supplementary Analysis 4: Hosmer-Lemeshow test and plots for the logistic regression models with covariates ZNF296 and KLF2.

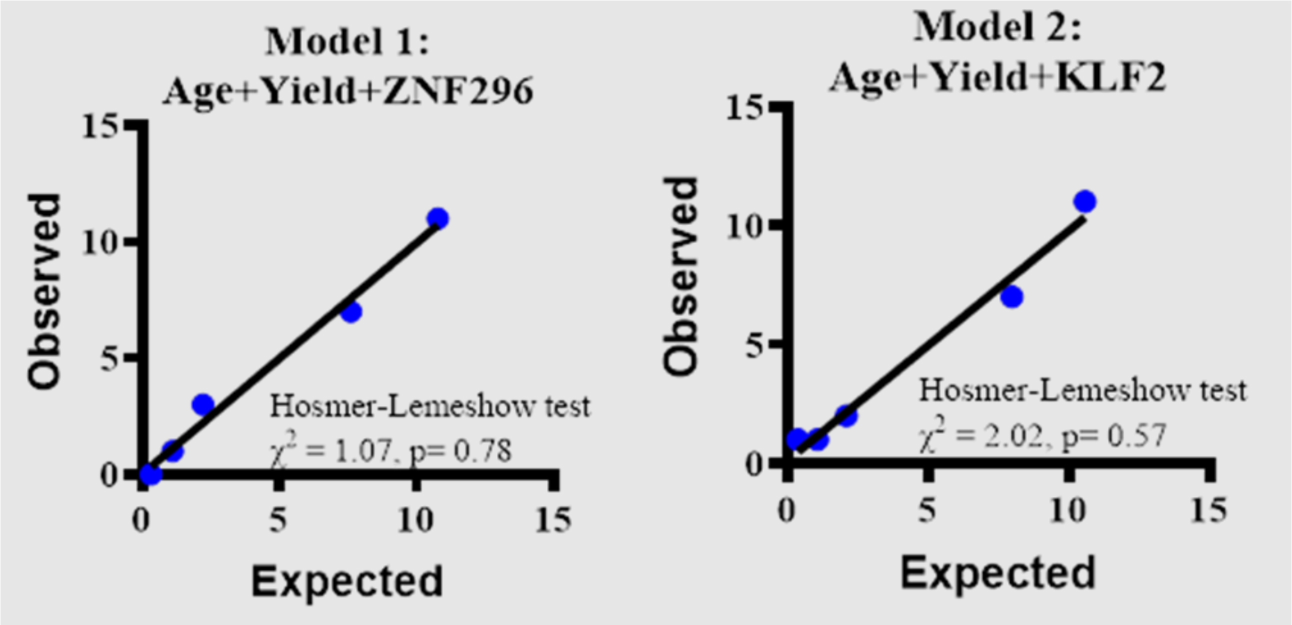

Supplementary figure 4: Hosmer-Lemeshow goodness of fit test was computed to assess model fit. Both models showed no evidence of poor model fit.

Supplementary Analysis 5: Bootstrap Analysis

Bootstrap result for the best fit Models revealed that the bootstrap standard errors of the estimates of ZNF296 and KLF2 (2.22 and 2.52 respectively) were like those obtained in the standard logistic regression model (2.5 and 2.48) and the coefficient estimates (-6.56 and -6.24) were within the 95% CI of the bootstrap estimates.

ZNF296	**Estimate**	**SE**	**95% CI**
β_{MLE}	-6.56	2.50	[-12.45, -2.36]
β_{boot}	-4.72	2.22	[-9.99, -1.40]
KLF2	**Estimate**	**SE**	**95% CI**
β_{MLE}	-6.24	2.48	[-11.78, -1.87]
β_{boot}	-4.52	2.52	[-9.73, -0.73]
